# Turning Informal Thesauri Into Formal Ontologies: A Feasibility Study on Biomedical Knowledge re-Use

**DOI:** 10.1002/cfg.247

**Published:** 2003-02

**Authors:** Udo Hahn

**Affiliations:** Text Knowledge Engineering Laboratory Natural Language Processing Division Albert-Ludwigs-Universität Freiburg Werthmannplatz 1 Freiburg D-79085 Germany

## Abstract

This paper reports a large-scale knowledge conversion and curation experiment. Biomedical domain knowledge from a semantically weak and shallow terminological resource, the UMLS, is transformed into a rigorous description logics format. This way, the broad coverage of the UMLS is combined with inference mechanisms for consistency and cycle checking. They are the key to proper cleansing of the knowledge directly imported from the UMLS, as well as subsequent updating, maintenance and refinement of large knowledge repositories. The emerging biomedical knowledge base currently comprises more than 240 000 conceptual entities and hence constitutes one of the largest formal knowledge repositories ever built.


**Comparative and Functional Genomics**
*Comp Funct Genom* 2003; **4**: 94–97.
Published online in Wiley InterScience (www.interscience.wiley.com). **DOI:** 10.1002/cfg.247

**Conference Paper**

### Turning informal thesauri into formal ontologies: a feasibility study on biomedical knowledge re-use

Udo Hahn*
*Text Knowledge Engineering Laboratory, Natural Language Processing Division, Albert-Ludwigs-Universität Freiburg, Werthmannplatz 1, D-79085 Freiburg, Germany*

**Correspondence to: Udo Hahn, Text Knowledge Engineering Laboratory, Natural Language Processing Division, Albert-Ludwigs-Universität Freiburg, Werthmannplatz 1, D-79085 Freiburg, Germany. E-mail: hahn@coling.uni-freiburg.de*

Received: 15 November 2002
Accepted: 3 December 2002

### Abstract

**This paper reports a large-scale knowledge conversion and curation experiment. Biomedical domain knowledge from a semantically weak and shallow terminological resource, the UMLS, is transformed into a rigorous description logics format. This way, the broad coverage of the UMLS is combined with inference mechanisms for consistency and cycle checking. They are the key to proper cleansing of the knowledge directly imported from the UMLS, as well as subsequent updating, maintenance and refinement of large knowledge repositories. The emerging biomedical knowledge base currently comprises more than 240 000 conceptual entities and hence constitutes one of the largest formal knowledge repositories ever built. Copyright © 2003 John Wiley & Sons, Ltd.**

**Keywords:** knowledge engineering; ontology engineering; biomedical knowledge; UMLS; description logics; formal reasoning; knowledge re-use; knowledge integration

### Introduction

Tasks such as disease encoding, searches for biomedical documents in bibliographic data bases, or the functional annotation of gene sequences usually require reference to shared domain knowledge. Typically, this sort of shared knowledge is made available through nomenclatures (controlled vocabularies), thesauri or classification codes. They serve the need to unify the use of lexical or phrasal variants of a single concept (by reference to a preferred term), to link terms via semantic relations [e.g. X is a broader or narrower term than Y, X is synonymous to Y, X is part of (or has part) Y], or to create a hierarchical system of increasingly specific categories, ordered according to decreasing generality of the terms and expressions they stand for. Biology and medicine, in particular, have a long-standing tradition in structuring their domain knowledge with the help of such 'documentation languages'. While they usually excel in a broad coverage of their domain (anatomy, pathology, pharmacology, genomics, etc.), their semantic foundations are weak. The interpretation of the terms or categories they provide, for instance, is still left to the intuition of an individual user whose view on the domain might differ from the views of others. In addition, specification gaps and inconsistencies are almost unavoidable, and their level of expressiveness is quite restricted (e.g. broader term, narrower term, synonymous term, related term).

While these terminological resources have proved to be useful already for tasks with humans playing a prominent role in the loop, they have almost never been considered for re-use in a fully computational environment in which problem solving, decision support or natural language understanding systems are embedded (some exceptions are due to Pisanelli *et al.*, 1998; Spackman, 2001). The formal encoding of domain knowledge (knowledge engineering) in this framework relies on knowledge representation languages that have their

Copyright © 2003 John Wiley & Sons, Ltd.

roots in various restricted forms of first-order logics (such as description logics) or semantic network formalisms (e.g. conceptual graphs; for a survey, see Sowa, 1991). These formal systems come with rigid, i.e. formally specified, semantics; they offer an enhanced level of expressiveness and sophisticated ways to encode conceptual restrictions or integrity constraints in order to guarantee sound and valid knowledge bases. Besides these semantic considerations the most outstanding difference between formally weak and strong approaches lies in the supply of reasoning engines, e.g. the classifier in description logics, which computes whether a concept is more specific than another one, based on a subsumption relation holding between them. The Janus face of formal knowledge engineering exhibits tremendous modelling efforts and high maintenance costs for the emerging knowledge bases. As a consequence, almost all of the domain descriptions built on formal approaches provide only quite a small coverage of the domain.

Our approach tries to combine the best of both worlds (for a more detailed presentation, see Hahn and Schulz, 2003). In essence, we intend to preserve all the benefits of formal knowledge representation approaches (the high level of conceptual expressiveness and integrity preservation, as well as the availability of formal reasoning devices, in particular), while we strive for maximum coverage of the domain. We cope with this challenge by converting the knowledge that has already been assembled in semantically weak terminological sources into a semantically stronger formal reasoning framework.

### Materials and methods

The terminological source we use is the Unified Medical Language System (UMLS; McCray and Nelson, 1995). It can be envisaged as an umbrella system, which covers more than 60 alternative medical thesauri and classification systems, such as MeSH, ICD, SNOMED and Digital Anatomist. From a conceptual perspective, the UMLS is divided into two major parts. The UMLS Semantic Network (SN), on the one hand, forms the upper ontology and consists of 134 semantic types linked by 54 types of semantic relations (7473 edges in total). The UMLS Metathesaurus, on the other hand, contains 776 940 concepts, each of which is assigned to one or more UMLS SN types. These concepts are linked by semantic relations taken from the UMLS SN type repertoire, making up 11 138 000 semantic links in the 2002 release. The vast majority of these links introduce thesaurus-style broader/narrower term relationships. For our experiments, we only considered the anatomy and pathology part of the UMLS, with 38 059 and 50 087 concepts involved, respectively.

The target to which knowledge from the UMLS is mapped is given by a (formally parsimonious) subclass of description logics, usually referred to as ALC. This language allows the definition of concepts by way of conjunction, disjunction and negation of concepts, and the definition of relations between concepts, which can be constrained by universal and existential quantification (for a formal definition, see Schmidt-Schauss and Smolka, 1991). Technically, we implemented the emerging knowledge base using the LOOM knowledge representation language (MacGregor and Bates, 1987).

The knowledge conversion workflow consists of four distinct steps:

1. Terminological axioms at the level of description logics are *automatically* generated from the relational table structures imported from the UMLS source. While all of the domain concepts from its anatomy and pathology section were taken into account, only a carefully selected subset of relation types from the UMLS were incorporated. Among those were relations such as *part-of/has-part, is-a* or *has-location*, since we considered them as reliable indicators for partonomic and taxonomic hierarchies, as well as spatial knowledge, respectively. We excluded, however, overly general ones such as *sibling-of* or *associated-with* from further consideration at this level of processing, since they are likely to introduce noise into the relational structure of the emerging knowledge base.
2. The 'raw' knowledge base is then immediately checked *automatically* by the description logics classifier (for details, see MacGregor, 1994) to see whether it contains definitional cycles and inconsistencies.

Copyright © 2003 John Wiley & Sons, Ltd.

3. If inconsistent or cyclic knowledge structures are encountered, a biomedical domain expert resolves the inconsistencies or cycles *manually*. After that, the classifier has to be rerun in order to check whether the modified knowledge base is consistent and non-cyclic with the changes made. A valid knowledge base at that level directly reflects the (still shallow) expressiveness of UMLS within a proper formal framework.
4. For many applications, the completeness and granularity (level of specificity) of UMLS specifications will not be sufficient. Hence, the knowledge base needs additional *manual* curation. Here we incorporate those relations that were not taken into consideration in previous rounds (e.g. *sibling-of* or *associated-with*) as a heuristic support for knowledge re-modelling, while also entirely new, quite specific relations are created (e.g. *inflammation-of, perforation-of* or *linear-division-of*). The latter are needed for deep automatic knowledge extraction from medical narratives, our major application (Hahn *et al*., 2002).

### Results and discussion

For the anatomy domain, we identified 1 cycle and 2328 inconsistent concept definitions, while for the pathology domain 355 cycles and not a single inconsistency occurred. Cycles and inconsistencies were removed manually, mainly by disabling relational links that were judged as unreasonable.

Our experimental evidence for updating and refining the emerging knowledge base in terms of more adequate and richer knowledge is currently still based on rather weak empirical foundations. We drew a random sample of 100 anatomy and pathology concepts for each domain. We have preliminary evidence that particularly knowledge-heavy relations, such as ‘pathological phenomenon X has anatomical location Y’, are subject to highly erroneous encoding in the UMLS (358 out of 522 relations were wrong, i.e. 69%). Conceptually demanding partonomic relations (part-of, has-part) as well as simpler taxonomic relations (is-a) have only few erroneous encodings but still may profit from the re-use of relations which have not been fed into the fully automatic process of knowledge base creation in the first round. Further manual enhancement (without any evidence from UMLS) is to a lesser extent required for taxonomic relations, although to a higher extent (factor 2) for partonomic ones.

Finally, we came up with one of the largest description logics knowledge bases ever built. Its size amounts to 164 000 concepts and 76 000 relations. The methodology we propose requires weak knowledge sources, such as thesauri, to be available. Only then may our approach serve as an alternative to developing domain knowledge bases from scratch (as evidenced by the GRAIL/GALEN experience, which resulted in a knowledge base finally composed of 9800 concepts; see Rector *et al*., 1997).

The easy part of knowledge conversion relates to the mapping task proper. Once a formal knowledge base has been set up, cleansing activities have to be undertaken in order to get rid of inconsistencies, specification gaps or granularity biases (often due to different terminological sources). In our framework, the availability of the description classifier turned out to be of utmost importance and outstanding heuristic value, since it helps to identify and to focus on the inconsistent portions of the emerging knowledge base.

Abstracting away from the particularities of our approach, some more general methodological problems for knowledge conversion and knowledge curation of this sort arise:

1. *Knowledge integration*. When several knowledge sources have to be combined, some knowledge portions may be overlapping and others may be far too distant, so that appropriate conceptual bridges have to be defined. Even knowledge sources that complement each other nicely require suitable interfaces, so that transition from one to the other is possible.
2. *Granularity*. Different knowledge sources, sometimes even a single one, often exhibit subdomain descriptions that are very fined-grained, as opposed to ones that are treated with much less specificity. Mediating between those different granularity levels of knowledge representation becomes an important requirement for adequate knowledge use. In addition, it might become necessary to provide intentionally different abstraction levels for the description of a single subdomain.
3. *Views*. There is no single, canonical view on particular domain knowledge. A tumour, for

Copyright © 2003 John Wiley & Sons, Ltd. 

instance, is at the same time an anatomical structure and a pathological phenomenon. This kind of ambiguity has immediate implications for its conceptual representation and the inferences derivable therefrom. Even more, alternative schools of thought differ in the way they organize the same subdomain. Hence, one has to provide formal devices that support different conceptual views on the same subject matter, rather than enforcing consensus in a dogmatic way.

4. *Top-level ontology*. The attempt to integrate the subdisciplines of a large science domain, such as biology or medicine, by a unifying ontological umbrella inevitably leads to the need to structure the abstract, 'upper' part of the underlying conceptual system. At this level, high-level concepts, such as 'organism' (e.g. animal, plant, virus), 'process' (photosynthesis, digestion), 'substance' (chlorophyll, blood) or 'structure' (animal or plant anatomy, cell morphology, etc.), have to be properly organized and conceptually represented so that they link to the more specific, concrete domain descriptions in a valid way.

### Acknowledgements

The paper could not have been written without the invaluable support of Stefan Schulz, who contributed his biomedical expertise and conducted the experiments.

### References

Hahn U, Romacker R, Schulz S. 2002. MedSynDiKATe: a natural language system for the extraction of medical information from finding reports. *Int J Med Inform* **67**(1–3): 63–74.

Hahn U, Schulz S. 2003. Towards a broad-coverage biomedical ontology based on description logics. In *Pacific Symposium on Biocomputing 2003*. World Scientific: New Jersey: 577–588.

MacGregor R. 1994. A description classifier for the predicate calculus. In *Proceedings of the 12th National Conference on Artificial Intelligence 1994*. AAI Press and MIT Press: Meulo Park, CA. 213–220.

MacGregor R, Bates R. 1987. *The LOOM Knowledge Representation Language*. Technical Report RS-87–188. Information Sciences Institute, University of Southern California.

McCray A, Nelson A. 1995. The representation of meaning in the UMLS. *Methods Inf Med* **34**(1/2): 193–201.

Pisanelli D, Gangemi A, Steve G. 1998. An ontological analysis of the UMLS Metathesaurus. In *Proceedings of the 1998 AMIA [American Medical Informatics Association] Annual Fall Symposium*. Hanley and Belfus: Philadelphia, PA. 810–814.

Rector A, Bechhofer S, Goble C, *et al*. 1997. The GRAIL concept modelling language for medical terminology. *Artif Intell Med* **9**: 139–171.

Schmidt-Schauss M, Smolka G. 1991. Attributive concept descriptions with complements. *Artif Intell* **48**(1): 1–26.

Sowa JF. 1991. *Principles of Semantic Networks. Explorations in the Representation of Knowledge*. Morgan Kaufmann: San Mateo, CA.

Spackman KA. 2001. Normal forms for description logic expression of clinical concepts in SNOMED RT. In *Proceedings of the 2001 AMIA [American Medical Informatics Association] Annual Fall Symposium*. Hanley and Belfus: Philadelphia, PA. 627–631.

Copyright © 2003 John Wiley & Sons, Ltd.

## References

[PDF_00312] Hahn U., Schulz S. (2003). Towards a broad-coverage biomedical ontology based on description logics.. Pac Symp Biocomput.

[PDF_00309] Hahn Udo, Romacker Martin, Schulz Stefan (2002). MEDSYNDIKATE--a natural language system for the extraction of medical information from findings reports.. Int J Med Inform.

[PDF_00323] McCray A. T., Nelson S. J. (1995). The representation of meaning in the UMLS.. Methods Inf Med.

[PDF_00325] Pisanelli D. M., Gangemi A., Steve G. (1998). An ontological analysis of the UMLS Metathesaurus.. Proc AMIA Symp.

[PDF_00330] Rector A. L., Bechhofer S., Goble C. A., Horrocks I., Nowlan W. A., Solomon W. D. (1997). The GRAIL concept modelling language for medical terminology.. Artif Intell Med.

[PDF_00338] Spackman K. A. (2001). Normal forms for description logic expressions of clinical concepts in SNOMED RT.. Proc AMIA Symp.

